# Comparative Transcriptomics Reveal Key Genes and Pathways Driving the Diversity of Heritable Inner Shell Nacre Colors in the Freshwater Pearl Mussel (*Sinohyriopsis cumingii*, Lea 1852)

**DOI:** 10.3390/ijms262211087

**Published:** 2025-11-16

**Authors:** Li Yuan, Zhiyan Wang, Min Zhang, Tingting Lu, He Wang, Xuefeng Lv, Jiale Li, Zhiyi Bai

**Affiliations:** 1 Key Laboratory of Freshwater Aquatic Genetic Resources, Ministry of Agriculture and Rural Affairs, Shanghai Ocean University, Shanghai 201306, China; l_yuan@shou.edu.cn (L.Y.); zywang@shou.edu.cn (Z.W.); lutt0918@163.com (T.L.);; 2 Shanghai Collaborative Innovation Center for Cultivating Elite Breeds and Green-Culture of Aquaculture Animals, Shanghai 201306, China; 3 Freshwater Pearl Science and Technology Backyard, Shanghai Ocean University, Wuyi 321200, China

**Keywords:** *Sinohyriopsis cumingii*, saibo, transcriptome analysis, pigment, structural coloration, epigenetic modification

## Abstract

Pearl color is the primary determinant of its value, and the mantle donor tissue (saibo) used in pearl culture plays a critical role in color formation. To elucidate the molecular mechanisms underlying nacre color, we performed comparative transcriptomic analyses of saibo tissues from *Sinohyriopsis cumingii* displaying three representative phenotypes: white (W), purple (P), and golden (G). A total of 364 differentially expressed genes (DEGs) (102 upregulated and 162 downregulated genes) were identified in W vs. P. A total of 770 DEGs (467 upregulated and 303 downregulated genes) were identified in W vs. G. KEGG pathway analysis revealed significant differences in the expression of genes mainly involved in pigment-based and structural coloration, including amino sugar and nucleotide sugar metabolism (ko00520), cell adhesion molecules (ko04514), tyrosine metabolism (ko00350), ECM-receptor interaction (ko04512), and PI3K-Akt signaling pathway (ko04151). Subsequent integrative analyses across W vs. P and W vs. G groups identified 45 key regulatory genes, which were classified into four functional categories: extracellular matrix protein synthesis and biomineralization (e.g., *chit*, *silkmaxin*, *bmp2/7*, *profilin*, *perlucin2*), organic pigment metabolism (e.g., *tyr*, *typ*, *dbh*, *bco2*, *gst5*, *ldlr*, *cpox*, *pks-like 1*, *pks15*), metal ion metabolism and accumulation (e.g., *hip-like*, *fcr1*, *ferritin 2*), and epigenetic regulation (e.g., *metK*, *mbd4/6*, *mettl24/27*, *alkbh6*). Taken together, our findings reaffirm the complex nature of pearl coloration and reveal that structural coloration, pigment biosynthesis, and epigenetic modulation collectively shape nacre color formation, which paves the way for further functional elucidation of color-related genes and facilitates selective breeding practices in *S. cumingii*.

## 1. Introduction

The diversity of animal coloration is a striking and pervasive phenomenon in the animal kingdom. This variation in coloration offers crucial adaptive advantages in courtship, camouflage, predation, social communication, and other ecological roles [1]. Mollusks, especially bivalves, display rich and varied color patterns. Shell colors vary among species and individuals, ranging from pure white and pale yellow to deep purple and iridescent blue, with some showcasing intricate patterns of interwoven colors [2]. This diversity in coloration is not only vital for ecological and evolutionary studies but also plays a key role in the market economy, significantly influencing consumer purchasing decisions [3,4]. Bivalves serve as a vital source of high-quality protein, essential vitamins, and minerals in the human diet, while their diverse shapes and vibrant shells are prized as natural decorative items [5,6]. Furthermore, pearls cultured from pearl oysters, offered in a spectrum of colors including white, pink, gold, emerald green, purple, and black, are highly sought after by consumers [7].

The formation of color diversity in bivalve shells and pearls is a complex process. The mantle, as the primary tissue responsible for shell and pearl formation, plays a crucial role in biomineralization and color development [8,9]. Unlike the chameleon, which has specialized pigment cells that rapidly respond to neural signals, bivalve mantles lack these specialized pigment layers [10]. Instead, color formation in their shells and pearls is a gradual process of secretion and accumulation. Through prolonged pigment deposition and integration with calcium carbonate crystals, a range of colors and patterns is ultimately displayed [2]. The formation of color diversity in shells and pearls is generally believed to primarily involve two mechanisms: pigment-based coloration, determined by pigment substances like carotenoids, polyphenols, and porphyrins, among others [8,11,12,13]. The deposition and distribution of different pigment types produce various colors and patterns. The other is structural coloration, arising from the interference, scattering, and diffraction effects caused by micro- or nanoscale structures, giving the shell or pearl a unique metallic sheen or iridescence [2,14,15]. These two mechanisms may act independently or synergistically, endowing the shell and pearl with rich colors and diverse optical properties.

Over the past three decades, numerous genetic breeding studies have highlighted the high heritability of shell color variations in bivalves. For example, a diallel hybridization study on golden, white, and black oysters showed that shell color is influenced by two genetic loci: one regulating pigment secretion and the other determining pigment distribution [16,17]. In *Macoma balthica*, shell colors—white, yellow, orange, and red—are determined by four alleles at a single locus, following a dominance hierarchy of red > orange > yellow > white [18]. In *Chlamys nobilis*, orange-purple dominates over both purple and brown, while purple dominates over brown. The orange gene also demonstrates an “epistatic effect” [19]. Overall, shell color has emerged as a key breeding objective for economically significant bivalves. Understanding the molecular basis of shell color variation is crucial for the molecular breeding of bivalves. Transcriptome sequencing has greatly advanced research in this area, enabling significant progress in uncovering the molecular mechanisms underlying shell color variation [8,20,21,22,23].

The freshwater pearl mussel (*Sinohyriopsis cumingii*) is a key species in pearl aquaculture, accounting for over 90% of global pearl production [24]. The mussel’s inner shell nacre displays a wide range of colors, with individuals from different color strains providing graft tissues (saibo) for cultivating pearls of corresponding colors [8,25,26,27,28]. This unique pigmentation mechanism makes the mussel an ideal model for investigating the molecular regulation of shell color polymorphism and pearl coloration in bivalves. Analyzing saibo tissues from different shell color strains enables a comprehensive understanding of the molecular basis underlying pearl color formation. Therefore, the current study selected three specific mussel populations with stable inheritance of white, purple, and golden inner shell nacre color. Their saibo tissues were collected for comparative transcriptomic analysis. The primary aim was to delve into the molecular landscape of pearl color variation. This approach provides a more comprehensive understanding of how different pearl colors emerge and may contribute to advancements in the molecular control of shell or pearl coloration.

## 2. Results

### 2.1. High Quality Transcriptomic Data

**Figure 1:**
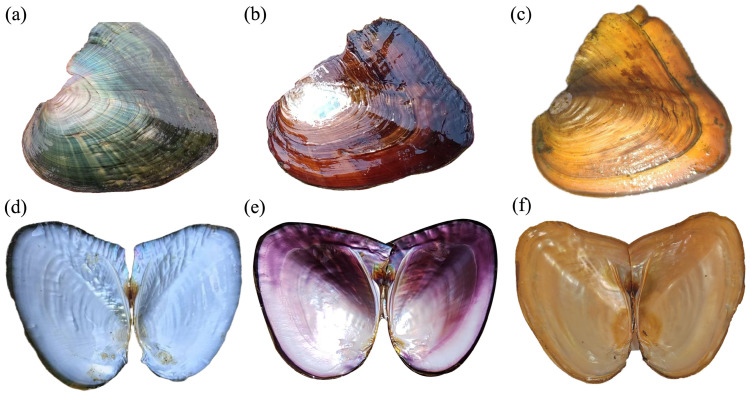
The visual appearance of three selectively bred *Sinohyriopsis cumingii* strains is shown from left to right: “Shenzhe No. 3” (**a**,**d**), “Shenzi No. 1” (**b**,**e**), and the golden strain (**c**,**f**). The top row shows their external shell color, and the bottom row displays their inner shell nacre color.

Mussels with typical inner shell colors were selected and sacrificed for analysis. Their external and inner shell colors are shown in Figure 1. A total of 372.90 million clean reads were obtained from nine libraries, with raw sequencing data per sample ranging from 5.63 Gb to 6.94 Gb. The genome mapping rate exceeded 85% for all samples, with an average of 87.30%. The average Q30 score (base error probability < 0.1%) across the nine samples was 95.32%, confirming the high quality of sequencing data and ensuring reliability for downstream analyses (Table 1).

### 2.2. Differentially Expressed Genes (DEGs) in Two Comparisons: W vs. P and W vs. G

**Figure 2:**
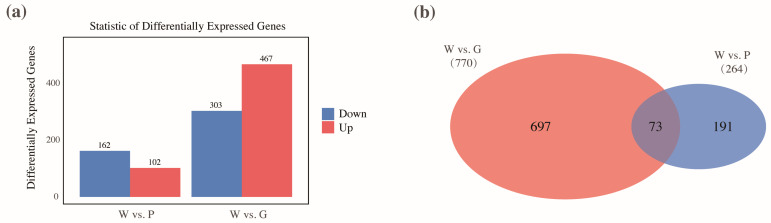
The expression patterns and distribution of differentially expressed genes (DEGs) differed among different comparison groups. (**a**) The number of upregulated and downregulated DEGs in the W vs. P and W vs. G comparison groups. (**b**) The overlap of DEGs between the W vs. P and W vs. G comparison groups.

**Figure 3:**
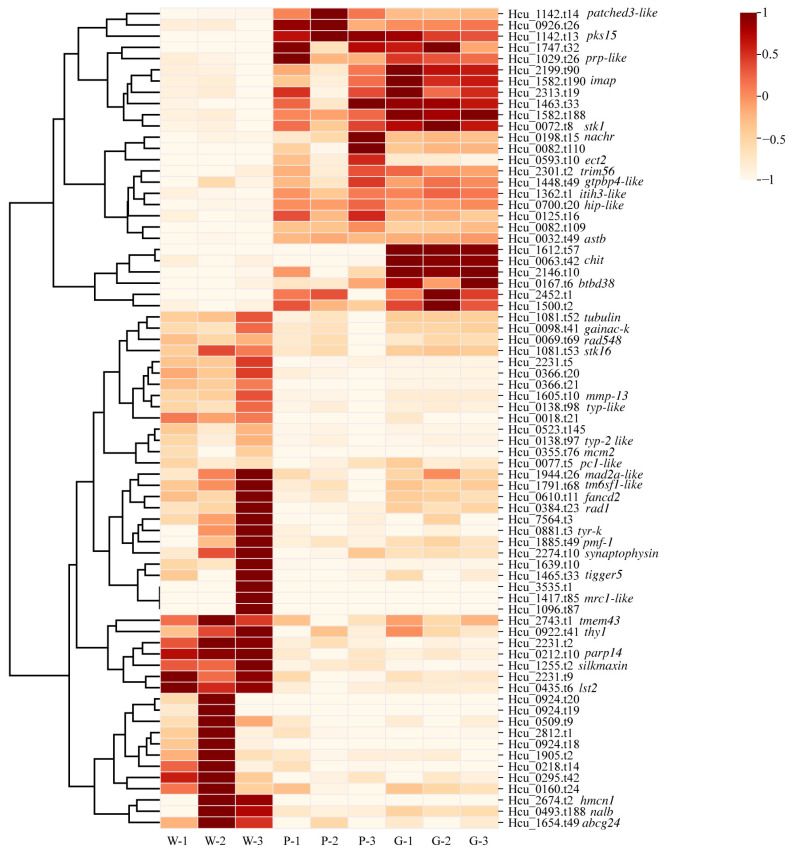
The expression patterns of 73 shared genes in the W vs. P and W vs. G comparisons are shown. Sequence identifiers and gene annotations for these genes are listed on the right side; genes without annotation names indicate that they remain uncharacterized. The FPKM value of gene expression was normalized using Min-Max Normalization. The specific formula used for scaling values to the [−1, 1] range is as follows: $x_{norm}\;$ = 2 × (x−min(x))/(max(x)−min(x)) – 1.

In the comparison group of W vs. P, a total of 264 DEGs were identified relative to the control group (W). Among these, 102 genes were significantly upregulated, while 162 genes were downregulated (Figure 2a). In contrast, the W vs. G group identified a greater number of DEGs, with 467 genes significantly upregulated and 303 genes downregulated (Figure 2a). Among the identified genes related to inner shell nacre color formation, 697 genes were unique to the comparison between W vs. G, while 191 genes were specific to the W vs. P group. Notably, a total of 73 genes were shared between the two comparison groups (Figure 2b). The expression profiles of these 73 genes were further analyzed across the W, P, and G groups. As shown in Figure 3, most genes exhibited similar expression patterns in the P and G groups, both significantly lower than W group, including genes such as tyrosinase-like protein (*typ like*), tyrosinase-like protein 2 (*typ-2 like*), serine/threonine-protein kinase 16 (*stk16*), *tubulin*, collagenase 3-like (*mmp-3*), *silkmaxin*, and macrophage mannose receptor 1-like (*mrc1-like*). Additionally, compared to W, genes including polyketide synthase type I Pks15 (*pks15*), patched domain-containing protein 3-like (*patched3-like*), pathogen-related protein-like (*prp-like*), GTPase IMAP family member (*imap*), serine/threonine-protein kinase 1 (*stk1*), neuronal acetylcholine receptor subunit beta-4-like (*nachr*), and chitotriosidase-1-like (*chit*) were significantly upregulated in both P and G groups, with *pks15*, *patched3-like* higher in P, and *imap*, *stk1*, and *chit* higher in G (Figure 3). Overall, these 73 shared genes were closely associated with the regulation of physiological metabolic processes, including pigment synthesis, lipid metabolism, metal ion metabolism, and biomineralization.

### 2.3. GO Term and KEGG Pathway Enrichment Analysis

**Figure 4:**
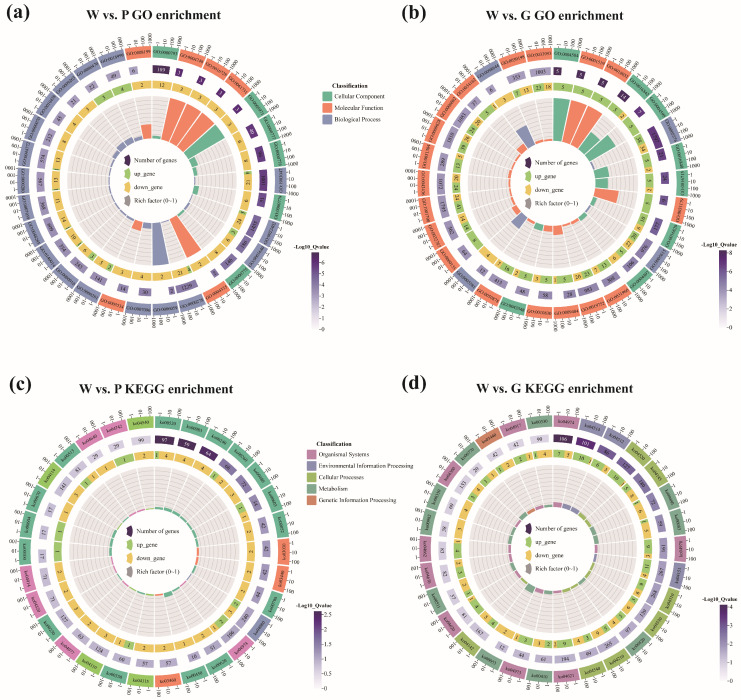
These circular plots show GO and KEGG enrichment for W vs. P (**a**,**c**) and W vs. G (**b**,**d**). Moving inside: the 1st circle illustrates the GO/KEGG categories, colored according to different functional categories. The 2nd circle illustrates the number of DEGs matched in the term, and the color depth denotes the degree of correlation. The 3rd circle displays upregulated (green) and downregulated (yellow) DEGs. The 4th (innermost) circles represent the Rich Factor values for different GO terms, with each cell of the background helpline representing 0.1.

To better understand the functional relevance of the DEGs, GO term and KEGG pathway enrichment analysis were performed. As shown in Figure 4a and Table S1, most GO terms in W vs. P group were closely associated with DNA synthesis and repair, cell cycle regulation, cellular stress responses, etc., such as condensed chromosome (GO:0000793), ribonucleoside-diphosphate reductase activity (GO:0004748), ribonucleoside-diphosphate reductase activity (GO:0061731), mitotic cell cycle process (GO:1903047) and mitotic cytokinesis (GO:0000281). In addition, the GO term “oxidoreductase activity, acting on CH or CH_2_ groups, disulfide as acceptor” (GO:0016728) was closely associated with organic biomacromolecules containing hydrocarbon structures, such as carotenoids. Moreover, the GO term “ferric iron binding” (GO:0008199) is related to the regulation of iron ion metabolism, whereas more complex GO terms were enriched in the W vs. G comparison group (Figure 4b and Table S2). For example, GO terms related to protein amidation and the modification of amino acids and their derivatives include “peptidylglycine monooxygenase activity” (GO:0004504), “protein amidation” (GO:0018032), and “amidine-lyase activity” (GO:0016842), etc. GO terms related to extracellular matrix proteins, which were crucial components of biomineralization, such as “extracellular region” (GO:0005576), “collagen trimer” (GO:0005581), and “extracellular matrix organization” (GO:0030198), etc. were enriched. Additionally, GO terms associated with carboxylic acid metabolism, crucial in synthesizing bio-organic pigments, were also significantly enriched. These included “carboxylic acid metabolic process” (GO:0019752), “phosphatidylinositol 3-kinase binding” (GO:0043548), “monocarboxylic acid metabolic process” (GO:0032787), and “organic acid metabolic process” (GO:0006082).

In alignment with the GO term enrichment results above, several metabolism pathways were shared between the W vs. P and W vs. G comparison groups (Figure 4c,d; and Tables S3 and S4). These enriched pathways are crucial for cell proliferation, energy metabolism, membrane structure formation, and cellular homeostasis, and are extensively involved in synthesizing secondary metabolites, including various amino acids, lipids, and nucleotides. Notably, pathways such as amino sugar and nucleotide sugar metabolism (ko00520), glutathione metabolism (ko00480), and gap junctions (ko04540) are essential for intercellular communication, metabolic regulation, and antioxidant defense. They are also critical for the synthesis, stabilization, and biomineralization of extracellular matrix proteins and polysaccharides. Additionally, the shared tyrosine metabolism pathway (ko00350) is essential for melanin synthesis, a crucial pigment in the formation of color phenotype in organisms.

### 2.4. Key Genes Involved in Inner Shell Color Formation

**Figure 5:**
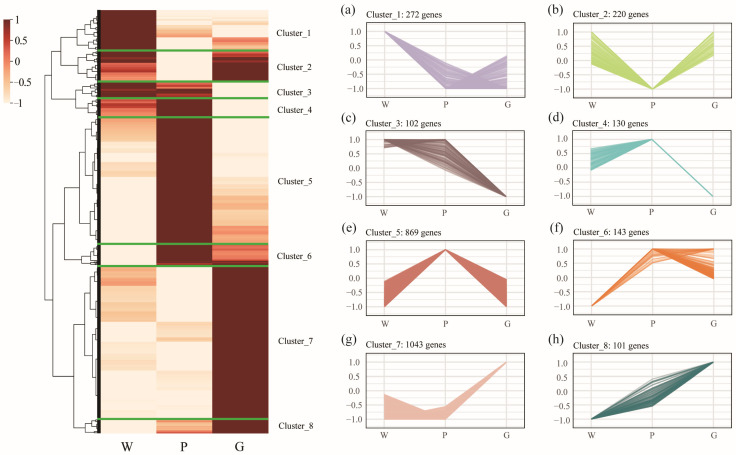
Hierarchical cluster analysis and expression profile of differentially expressed genes (DEGs) in the mussels with different inner shell color. The subfigures (**a**–**h**) on the right illustrate the expression patterns of the eight clustered gene sets across the W, P, and G groups. These DEGs represent the union of all differentially expressed genes identified across pairwise comparisons among the three mussel groups, comprising a total of 2880 genes. The FPKM value of gene expression was normalized using Min-Max Normalization. The specific formula used for scaling values to the [−1, 1] range is as follows: $x_{norm}\;$ = 2 × (x−min(x))/(max(x)−min(x)) – 1.

Limiting candidate gene identification for inner shell color formation to pairwise comparisons may overlook key expression trends across groups. To further investigate regulatory genes for inner shell color, we combined all DEGs from pairwise comparisons (union of all groups) and displayed their expression patterns in a heatmap. As a result, a total of 2880 DEGs were collected and divided into eight clusters, with the expression patterns of these clusters displayed in sideward line charts (Figure 5). The number of genes in each cluster varies, listed in descending order: cluster 7 (1043 genes), cluster 5 (869 genes), cluster 1 (272 genes), cluster 2 (220 genes), cluster 6 (143 genes), cluster 4 (130 genes), cluster 3 (102 genes), and cluster 8 (101 genes). The expression patterns of genes in clusters 3 and 4 were similar, showing no significant difference between groups W and P, while both were significantly higher than in group G. Genes in clusters 2 and 5 exhibited opposite expression patterns, with genes in cluster 2 displaying a high-low-high pattern across groups W, P, and G, whereas genes in cluster 5 showed a low-high-low pattern. Genes in clusters 6 and 8 were upregulated in groups P and G, with the highest expression in P for cluster 6 and in G for cluster 8. These results suggest that the formation of diverse shell color phenotypes is a complex process involving the coordinated regulation of multiple genes.

By integrating DEGs, functional annotation, GO, and KEGG enrichment, and the varied expression patterns of clustered genes across the three groups, forty-five key genes potentially involved in inner shell nacre color formation were identified (Table 2). These include 14 genes associated with extracellular matrix protein synthesis and biomineralization, 21 genes linked to organic pigments (e.g., melanins, carotenoids, porphyrins, and quinones), 4 genes related to metal ion metabolism, and 6 genes involved in epigenetic modification. Specially, the mRNA expression levels of *silkmaxin*, *mmp-19*, and *perlucin2* were highest in W, while *col6a*, *gal3st3-like*, *beta-hexosaminidase*, *hemicentin-1-like*, and *nidogen-2* were highest in P. Genes involved in the melanin metabolism pathway (e.g., *tyr*, *typ2-like*, *typ-like*, *dbh*) were highly expressed in W, whereas *slc12* was upregulated in P and G. The carotenoid synthesis and metabolism-related gene *bco2* showed high expression in W, *lrat* in G, and *gst5* in P. *ldlr* was highly expressed in both P and G, with the highest expression in G. The expression of the porphyrin metabolism-related gene *fech x1* was highest in P, followed by G, and lowest in W. In contrast, *pbgd* and *cpox* were more highly expressed in G, with no significant differences between P and W. Naphthoquinone-based pigments may also play an important role in shell coloration, with *pks-like 1* significantly upregulated in P and *msdh* significantly upregulated in G. Additionally, genes related to metal ion deposition and transport, such as *hip-like* (significantly expressed in both P and G) and *fcr1* and *chn1* (highly expressed in G), were also identified. Particularly noteworthy were the epigenetic modification-related genes *metK*, *mbd4*, *mbd6*, *mett124, mettl27*, and *alkbh6*. *metK* was significantly upregulated in G, while *mbd4*, *mbd6*, *mett124*, and *mettl27* were highly expressed in W; *alkbh6* was highly expressed in both P and G, with the highest expression observed in P.

### 2.5. qRT-PCR Validation

**Figure 6:**
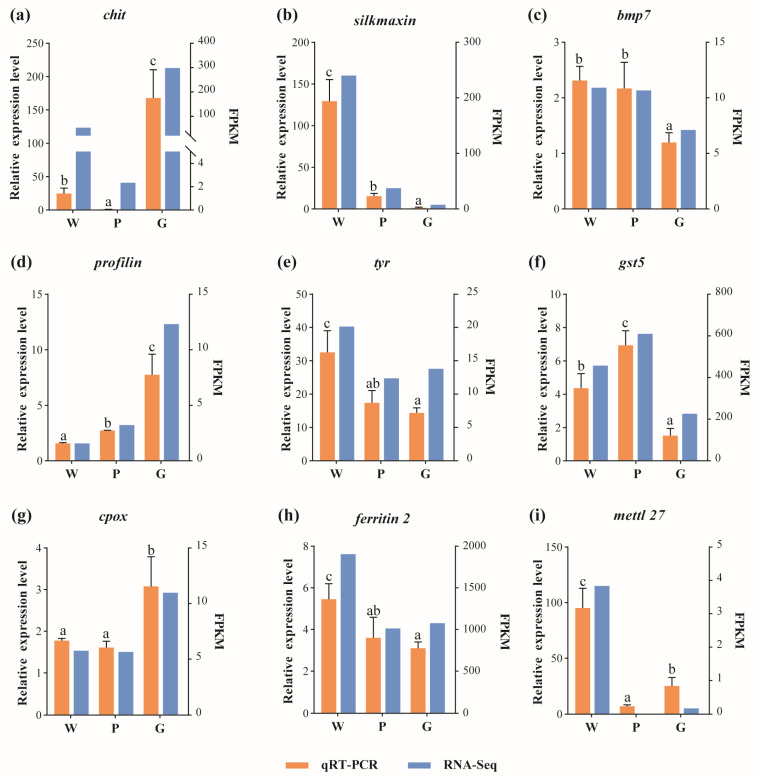
Validation of RNA-Seq expression patterns using qRT-PCR. Figures (**a**–**i**) represent the qRT-PCR data and corresponding transcriptome FPKM values for the *chit*, *silkmaxin*, *bmp7*, *profilin*, *tyr*, *gst5*, *cpox*, *ferritin 2*, and *mettl27* genes across the W, P, and G groups. qRT-PCR data were expressed as mean ± standard deviation (SD); bars with different letters differ significantly (*p* < 0.05). Homogeneity of variance was tested with Levene’s test. One-way analysis of variance (ANOVA) followed by Tukey’s post hoc test was performed to determine statistical differences among groups, with *p* < 0.05 considered statistically significant. All statistical analyses were performed using the SPSS statistics package software (version 26.0). RNA-Seq expression levels were presented as the mean FPKM values for each group.

The expression patterns of these nine randomly selected DEGs are shown in Figure 6. The qRT-PCR results confirmed that all nine selected genes exhibited expression patterns consistent with the RNA-Seq data, indicating the accuracy and high reliability of the RNA-Seq analysis.

## 3. Discussion

The mantle tissue is commonly selected for transcriptome sequencing and analysis when investigating the mechanisms underlying shell color polymorphism in bivalves. However, this approach has some limitations. Although the mantle is the primary tissue involved in shell formation, shell color may be influenced by the coordinated actions of other tissues, such as hemolymph, hepatopancreas, and different regions of the mantle. Analyzing only the mantle may not fully reveal the molecular mechanisms regulating shell color. The color of pearls is primarily determined by the gene expression of donor graft tissues (saibo), which directly participate in the formation of the pearl sac, the regulation of pearl nacre secretion, and the synthesis of pigment substance following transplantation [8,27]. Direct transcriptome analysis of saibo tissue allows for the capture of gene regulatory patterns related to pearl color formation, avoiding interference from the host individual or other tissues. Our findings suggest that the molecular regulatory network governing pearl color formation is intricate and multifaceted. By integrating multi-dimensional data, we identified four primary factors that influence pearl color: extracellular matrix protein synthesis and biomineralization, organic pigments (e.g., melanins, carotenoids, porphyrins, and quinone compounds), metal ion metabolism and accumulation, and epigenetic modifications.

The extracellular matrix (ECM) is an essential component of the extracellular environment, composed mainly of structural proteins (e.g., collagen, fibronectin, and silkmaxin), glycosaminoglycans, proteoglycans, and integrins. These components collaborate to support cellular structure, provide an initial organic framework for calcium carbonate deposition, and regulate the morphology and arrangement of crystals, thus significantly influencing the biomineralization process of shells and pearls [29,30]. However, recent studies have shown that several ECMs play a critical role in shell or pearl color formation, a phenomenon known as structural coloration [2,15]. During pearl formation, ECMs regulate the growth direction, size, and arrangement of aragonite crystals, which influence the refractive index differences and interlayer thickness of the nanoscale layered structure [31]. This process results in the manifestation of distinct colors through light reflection, diffraction, and interference. In this study, we identified several ECMs and observed significant expression differences across various inner shell nacre color strains in the *S. cumingii* population, further confirming that structural coloration is a key factor in pearl color diversity.

The synthesis, transport, and accumulation of pigment compounds are critical determinants of shell coloration in bivalves [2]. In white mussels (W), *tyr*, *typ2-like*, *typ-like*, and *dbh* showed higher expression levels than in purple (P) and golden mussels (G), while *slc12* was significantly upregulated in P. This expression pattern suggests that melanin biosynthesis in bivalves is regulated by a complex molecular network. Dopamine-β-hydroxylase (DBH) converts dopamine to norepinephrine, limiting dopamine availability for melanin synthesis [32], while *slc12* maintains intracellular Na^+^/K^+^/Cl^−^ balance and organelle pH, optimizing melanosomal conditions and promoting pigmentation [33]. Genes involved in carotenoid metabolism, transport, and storage also show significant differential expression. β-Carotene 9′,10′-oxygenase (*bco2*) shows the highest expression in W. BCO2 catalyzes the asymmetric cleavage of carotenoids, promoting pigment degradation and resulting in a lighter shell phenotype [34]. In G, *lrat* and *ldlr* are markedly upregulated, indicating enhanced esterification and storage of carotenoid derivatives, along with active lipoprotein-mediated uptake [35]. This facilitates pigment deposition in the mantle and shell, producing a golden hue. P shows high expression of *gst5* and *ldlr class B*, combined with lower expression of cleavage enzymes, suggesting that pigment accumulation occurs via GST-mediated carotenoid binding and stabilization, together with efficient receptor-mediated uptake. Overall, differential pigmentation among the three mussels can be summarized as a coordinated “cleavage (BCO2)—modification (CYP/LTD5)—uptake (LDLR)—binding/stabilization (GST)—esterification/storage (LRAT)” network. W is dominated by cleavage, resulting in pigment reduction; G relies on uptake and storage for enhanced golden deposition; P achieves strong coloration through synergistic binding and uptake mechanisms. In terms of porphyrin metabolism, *fech x1*, *pbgd*, and *cpox* show relatively low expression in W, potentially leading to insufficient porphyrin accumulation and a lighter shell color phenotype [36]. In P, *fech*—the terminal, rate-limiting enzyme converting protoporphyrin IX to heme—is markedly upregulated, suggesting enhanced heme biosynthesis and implicating iron-porphyrin derivatives in purple shell formation. In G, elevated *pbgd* and *cpox* indicate enhanced porphyrin synthesis and oxidation, with intermediate accumulation likely contributing to golden shell coloration [37]. Furthermore, genes involved in polyketide synthesis and naphthoquinone pigment production show marked differential expression among the three mussels. *pks-like 1* and *pks15* are strongly upregulated in P, indicating active polyketide and naphthoquinone pigment synthesis that likely serve as primary contributors to the purple shell phenotype. This is consistent with our previous GWAS findings on purple inner shell coloration [28]. Moreover, studies in echinoderms have shown that naphthoquinone pigments are essential for purple coloration. For example, *pks* knockout in sea urchins abolishes purple pigmentation and produces a white phenotype, directly linking naphthoquinone biosynthesis to purple coloration [38,39,40]. Together with our findings, the high expression of *pks* genes in P suggests that a similar naphthoquinone biosynthesis pathway likely underlies purple shell formation in mollusks.

Regarding metal ion homeostasis and transport. In W, *ferritin 2* was markedly upregulated, suggesting enhanced iron sequestration and reduced free iron availability, which may limit heme and porphyrin biosynthesis and contribute to the lighter phenotype [41]. *hip-like*, enriched in metal-binding motifs and capable of interacting with Fe, Cu, and Zn, plays a central role in ion transport and homeostasis [42]. Its strong upregulation in P and G suggests that enhanced metal-binding and transport capacity may facilitate metal-dependent pigment deposition. In G, the distinct upregulation of *fcr1* and *chn1*, which promote Fe^3+^ reduction to Fe^2+^ and mediate metal coordination, is particularly noteworthy, as Fe^2+^ is a direct substrate for heme and porphyrin biosynthesis and may drive golden shell formation [37].

Notably, we observed pronounced differences in the expression of genes associated with epigenetic modification among the three mussels. Epigenetic regulation—including DNA methylation, RNA methylation, and histone modifications—constitutes a critical “second layer” of gene expression control and plays a central role in the spatiotemporal regulation of pigment biosynthesis [43]. In this study, *metK* (encoding S-adenosylmethionine synthase) was highly expressed in G, indicating an enhanced capacity for methyl donor synthesis and providing substrates for extensive methylation. Conversely, *alkbh6* (a demethylase) showed elevated expression in both P and G, potentially conferring increased dynamic plasticity in pigment-related gene regulation. These findings indicate that shell coloration is influenced not only by metabolic and transport pathways but also by finely tuned epigenetic networks. Accumulating evidence from other bivalves—including *Crassostrea gigas* [44], *Patinopecten yessoensis* [45], *Pinctada fucata martensii* [46], and *Pinctada margaritifera* [47]—establishes epigenetic modification as a key regulatory mechanism of shell pigmentation. In particular, DNA methylation has been shown to modulate the expression of key melanogenic genes (e.g., *tyr*, *tyrp1*, and the microphthalmia-associated transcription factor *mitf*) and multiple paracrine factors (e.g., stem cell factor *scf* and endothelin-1 *et-1*), providing direct evidence that melanogenesis is tightly linked to methylation dynamics [48,49]. Together with our findings, this strongly suggests that epigenetic regulation exerts a broad and fundamental influence on shell color determination in bivalves. We propose that shell color divergence is likely established through the synergistic interplay between metabolic pathways and epigenetic modifications. In the future, integrative analyses combining methylome, transcriptome, and multi-layered epigenomic data will be crucial to fully elucidate the epigenetic mechanisms underlying shell color formation and to provide novel strategies for breeding programs aimed at shell color improvement.

## 4. Materials and Methods

### 4.1. Experimental Mussels

In mid-November 2022, healthy 3-year-old mussels with purple, white, and golden inner shell colors were selected from the F4 generation of “Shenzi No. 1”, the F3 generation of “Shenzhe No. 3” and the F4 generation of the golden strain, respectively. These mussels were used as broodstock and placed in three separate, algae-rich ponds at the Freshwater Pearl Science and Technology Backyard in Wuyi, Zhejiang Province. In April 2023, 24 females and 12 males with well-developed gonads were selected from each of the three ponds, and pairings were initiated in net cages at a ratio of four females to two males per cage. After 7 days, the development of fertilized eggs in the gills of female mussels was inspected. Once the glochidia matured, the females were removed, shade-dried for four hours, and placed in a hatchery tank to induce glochidia release. Yellow catfish (*Pelteobagrus fulvidraco*) were used as hosts to collect the glochidia. After 10 days, the glochidia detachment rate in the gills of the catfish was checked. Once the detachment rate exceeded 80%, all catfish were removed. Detailed descriptions of the subsequent two stages of daily culture management—indoor greenhouse cultivation and outdoor pond rearing—can refer to our previous publication [50]. Importantly, both breeding and juvenile rearing were conducted under identical environmental conditions to minimize external influences.

After 7 months, individuals with typical inner shell colors were selected from the progeny of the three strains. External and inner shell colors were recorded, and the edge mantle tissues were collected. Skilled nucleation technicians carefully removed the outer membrane of the edge mantle tissue (known as the saibo), which was immediately stored in liquid nitrogen for RNA extraction. Nine samples were collected from each group, with every three samples pooled to form a single biological sample, resulting in three biological replicates per group.

### 4.2. RNA Extraction, cDNA Library Construction, and Sequencing

Total RNA was extracted from the saibo tissue using RNAiso Plus Reagent (Takara, Dalian, China) according to the manufacturer’s instructions. The total RNA quality and purity were estimated using agarose gel electrophoresis and NanoDrop 2000 (Thermo Fisher Scientific Inc., Wilmington, DE, USA), respectively. High-quality total RNA samples (OD260/280 ranged 1.8–2.2, RIN ≥ 7.5) were retained. The mRNA, enriched from total RNA using poly-T oligo-attached magnetic beads, was used to synthesize the first-strand cDNA with random oligonucleotides and SuperScript II. Subsequently, the second-strand cDNA was synthesized using DNA Polymerase I and RNase H. The 3′ ends of the DNA fragments were then adenylated, followed by ligation of Illumina PE adapter oligonucleotides for hybridization. Finally, cDNA fragments of the preferred length (400–500 bp) were purified using the AMPure XP system (Beckman Coulter, Beverly, CA, USA). DNA fragments with ligated adapters at both ends were selectively enriched using the Illumina PCR Primer Cocktail in a 15-cycle PCR reaction (Illumina, San Diego, CA, USA). The products were purified with the AMPure XP system and quantified using the Agilent high-sensitivity DNA assay on a Bioanalyzer 2100 system (Agilent, Technologies, Santa Clara, CA, USA). The sequencing library was subsequently sequenced on the NovaSeq 6000 platform (Illumina, San Diego, CA, USA), producing paired-end reads of 150 nucleotides.

### 4.3. Data Processing, Assembly, and Differentially Expressed Genes (DEGs) Analysis

Adapter-containing and low-quality reads were filtered out from the raw sequencing data generated by high-throughput sequencing using fastp (v 0.22.0) software to obtain clean reads. The clean reads were aligned to the *S. cumingii* reference genome GCA_028554795.2 using HISAT2 (v 2.1.0) [51]. HTSeq (v 0.11.2) was used to count the reads mapped to each gene to obtain raw expression levels, which were then normalized using FPKM (Fragments Per Kilobase of transcript per million mapped reads) [52]. Differentially expressed genes (DEGs) were analyzed using DESeq (v 1.38.3), with |log2FoldChange| > 1 and *p*-value < 0.05 as the thresholds for significant expression differences. Gene expression profiles were compared across three groups: mussels with pure white (W), deep purple (P), and bright golden (G) inner shell colors. All DEGs from each comparison (W vs. P and W vs. G) were submitted for GO functional and KEGG pathway enrichment analysis using the ClusterProfiler R package (v 4.1.0) [53]. Specifically, enrichment significance was calculated using a hypergeometric test, and the resulting *p*-values were adjusted for multiple comparisons using the Benjamini–Hochberg method. Enriched terms with an adjusted *p*-value (*Q*-value) < 0.05 were considered statistically significant [54].

### 4.4. Validation of the DEGs by qRT-PCR

Nine genes associated with inner shell nacre color formation were randomly selected for validation via quantitative real-time PCR (qRT-PCR). Primer pairs were designed using Primer 5.0, and detailed primer information was presented in Table S5. The Bio-Rad-CFX-96 (Bio-Rad, Hercules, CA, USA) machine and SYBR RT-PCR Kit (RR420A, Takara) were used for qRT-PCR. The reaction system and PCR protocols followed previously described methods [28]. All measurements were conducted in triplicate, normalized to *EF-1α* expression, and analyzed using the 2^−ΔΔCt^ method.

## Figures and Tables

**Table 1:** Details of the transcriptomic data for each of the nine samples.

Sample	Raw Data (Gb)	Clean Reads (Mb)	Q30 (%)	Genome Mapping Ratio (%)
W-1	6.35	41.09	94.93	86.73
W-2	6.55	42.47	95.41	85.97
W-3	6.70	43.45	95.62	86.35
P-1	5.63	36.33	95.03	88.94
P-2	6.27	40.67	95.24	89.69
P-3	6.94	44.50	95.33	89.45
G-1	5.97	38.69	95.39	86.25
G-2	6.30	40.90	95.45	86.11
G-3	6.83	44.31	95.48	86.22

Note: W-1–W-3, P-1–P-3, and G-1–G-3 represent the three biological replicates of the white, purple, and golden shell groups, respectively.

**Table 2:** Summary of differentially expressed genes related to inner shell nacre color formation of *S. cumingii*.

Functional Category	Gene ID	Gene Name	FPKM		
			W	P	G
Extracellular matrix protein synthesis and biomineralization					
	Hcu_0063.t42	Chitinase 1, *chit*	52.90	2.33	298.23
	Hcu_1255.t2	*silkmaxin*	240.13	37.35	7.41
	Hcu_1605.t10	metalloproteinase-19, *mmp-19*	1543.82	126.86	373.84
	Hcu_0804.t25	Collagen alpha-6(VI) chain, *col6a*	1.11	7.41	2.01
	Hcu_0000.t94	Galactose-3-O-sulfotransferase 3-like, *gal3st3-like*	0.00	1.35	0.73
	Hcu_1654.t70	*beta-hexosaminidase*	0.86	5.24	3.25
	Hcu_1244.t82	Bone morphogenetic protein 2, *bmp2*	1.03	4.87	9.00
	Hcu_1016.t47	Bone morphogenic protein 7, *bmp7*	10.91	10.67	7.10
	Hcu_1169.t12	*hemicentin-1-like*	4.85	6.96	3.33
	Hcu_1421.t27	*profilin*	1.57	3.21	12.30
	Hcu_0426.t68	Ornithine decarboxylase, *odc*	1.32	4.66	9.41
	Hcu_0616.t60	*perlucin2*	69.04	49.67	13.89
	Hcu_1817.t2	Myosin Regulatory Light Chain 12A-like, *mrlc12a-like*	117.60	123.19	83.50
	Hcu_0501.t34	*nidogen-2*	138.74	152.74	103.43
Organic pigment (e.g., melanins, carotenoids, Porphyrins, Quinones)					
	Hcu_0138.t92	Tyrosinase, *tyr*	20.11	12.37	13.80
	Hcu_0138.t98	Tyrosinase-like protein 2, *typ2-like*	21.15	2.65	4.44
	Hcu_0138.t97	Tyrosinase-like protein, *typ-like*	14.35	1.27	1.44
	Hcu_1957.t20	Dopamine beta-hydroxylase, *dbh*	14.76	0.44	0.04
	Hcu_0050.t13	Solute carrier family 12, *slc12*	5.18	26.80	13.73
	Hcu_1214.t20	Beta-carotene 9′,10′-oxygenase, *bco2*	11.89	8.17	7.38
	Hcu_1266.t92	Lecithin retinol acyltransferase, *lrat*	1.02	1.29	10.48
	Hcu_0521.t37	Glutathione S-transferase 5, *gst5*	457.62	610.03	225.79
	Hcu_1784.t83	Lutein Deficient 5, *ltd5*	5.81	4.45	0.16
	Hcu_0686.t3	Low-density lipoprotein receptor-related protein 4-like, *ldlr*	1.05	3.54	24.62
	Hcu_2050.t33	Low-density lipoprotein receptor repeat class B, *ldlr class B*	84.53	95.48	62.08
	Hcu_0784.t110	Cytochrome P450, *cyp450-13a4*	10.03	10.74	3.29
	Hcu_1637.t6	*hsp70*	3.20	0.32	3.89
	Hcu_0198.t15	Neuronal acetylcholine receptor subunit beta-4-like, *nachr*	0.17	3.38	2.65
	Hcu_0435.t18	Ferrochelatase, mitochondrial-like isoform X1, *fech x1*	1.28	4.39	2.49
	Hcu_2474.t35	Porphobilinogen deaminase, *pbgd*	0.34	0.48	2.61
	Hcu_0662.t20	Oxygen-dependent coproporphyrinogen-III oxidase, *cpox*	5.74	5.63	10.97
	Hcu_0415.t17	Methylmalonate-semialdehyde dehydrogenase, *msdh*	0.57	1.69	4.30
	Hcu_1142.t12	Polyketide synthase-like 1, *pks-like 1*	0.16	5.49	0.69
	Hcu_1142.t13	Synthesis polyketide synthase type I, *pks15*	0.00	4.09	5.00
	Hcu_0119.t14	Quinone oxidoreductase, *qor*	31.10	27.54	22.23
Metal ion metabolism and accumulation					
	Hcu_0700.t20	Heavy metal-binding protein, *hip-like*	3.76	27.74	25.07
	Hcu_0398.t50	Ferric-chelate reductase 1, *fcr1*	0.02	0.08	1.11
	Hcu_0479.t45	Ferritin subunit 2, *ferritin 2*	1902.76	1010.76	1075.66
	Hcu_1235.t12	Cysteine and histidine-rich domain-containing protein 1, *chn1*	0.66	2.19	19.56
Epigenetic modification					
	Hcu_1546.t4	S-adenosylmethionine synthetase, *metK*	289.08	158.80	2313.67
	Hcu_0104.t4	Methyl-CpG-binding domain protein 6, *mbd6*	0.10	0.02	0.02
	Hcu_0214.t12	Methyl-CpG-binding domain protein 4, *mbd4*	0.15	0.08	0.02
	Hcu_1494.t46	Methyltransferase-like protein 24, *mettl24*	0.17	0.03	0.00
	Hcu_1698.t34	Methyltransferase-like protein 27, *mettl27*	3.83	0.00	0.17
	Hcu_0501.t138	Alpha-ketoglutarate-dependent dioxygenase alkB homolog 6, *alkbh6*	5.80	13.76	10.08

## Data Availability

Data will be made available on request. RNA sequencing raw reads have been deposited in NCBI’s Sequence Read Archive (SRA) under BioProject accession number PRJNA1334136.

## Supplementary Materials

The following supporting information can be downloaded at: https://www.mdpi.com/article/10.3390/ijms262211087/s1.
